# Anthropogenic host plant expansion leads a nettle-feeding butterfly out of the forest: consequences for larval survival and developmental plasticity in adult morphology

**DOI:** 10.1111/eva.12249

**Published:** 2015-02-13

**Authors:** Thomas Merckx, Mélanie Serruys, Hans Van Dyck

**Affiliations:** Behavioural Ecology and Conservation Group, Biodiversity Research Centre, Earth and Life Institute, Université catholique de LouvainLouvain-la-Neuve, Belgium

**Keywords:** *Aglais urticae*, eco-evolutionary dynamics, eutrophication, habitat expansion, host plant quality, phenotypic space, range expansion, rapid evolution, split-brood, *Urtica dioica*

## Abstract

Recent anthropogenic eutrophication has meant that host plants of nettle-feeding insects became quasi-omnipresent in fertile regions of Western Europe. However, host plant resource quality – in terms of microclimate and nutritional value – may vary considerably between the ‘original’ forest habitat and ‘recent’ agricultural habitat. Here, we compared development in both environmental settings using a split-brood design, so as to explore to what extent larval survival and adult morphology in the nettle-feeding butterfly *Aglais urticae* are influenced by the anthropogenic environment. Nettles along field margins had higher C/N ratios and provided warmer microclimates to larvae. Larvae developed 20% faster and tended to improve their survival rates, on the agricultural land compared to woodland. Our split-brood approach indicated plastic responses within families, but also family effects in the phenotypic responses. Adult males and females had darker wing pigmentation in the drier and warmer agricultural environment, which contrasts with the thermal melanism hypothesis. Developmental plasticity in response to this microclimatically different and more variable habitat was associated with a broader phenotypic parameter space for the species. Both habitat expansion and developmental plasticity are likely contributors to the ecological and evolutionary success of these nettle-feeding insects in anthropogenic environments under high nitrogen load.

## Introduction

Human-induced global environmental change consists of several interacting processes, such as habitat conversion and climatic change, driving the current biodiversity alterations and crisis (Brook et al. 2008; Barnosky et al. 2011). Except for organisms with pre-adaptations (e.g. high degree of phenotypic plasticity) to resist or exploit such changes (e.g. urban exploiters: Kark et al. 2007), these drivers represent major challenges, forcing populations to either perish or alter developmental characteristics, phenotypic trait expression and life-history strategies by rapid evolution and by spreading these evolutionary adaptations through dispersal (Reznick and Ghalambor 2001; Gilbert 2005; Sultan 2007; Saccheri et al. 2008; Sih et al. 2011). As such, the ability of populations to cope with changing, human-dominated environments is determined by their local genetic architecture and, more specifically, by the mobility and by the degree of phenotypic and developmental plasticity of their individuals (Gilbert 2001; Nijhout 2003).

Plastic responses to novel conditions have the potential to rapidly alter the targets of natural selection, and particularly so for behavioural responses, such as oviposition site selection (Sih et al. 2011). Species, populations and individuals show marked variation in behaviour and in degree of behavioural plasticity, which can influence the rate and outcome of adaptive evolution (e.g. Charmantier et al. 2008). Developmental plasticity may include trade-offs during development in resource allocation towards different adult traits, thus impacting on individual fitness, in response to the interplay of genetic and environmental conditions (Nijhout and Emlen 1998; Boggs 2009; Snell-Rood 2013). These trade-offs are especially pronounced in insects because juvenile ecological requirements differ profoundly from those at the adult stage (Speight et al. 2008). How specific environmental conditions affect larval development and the resulting resource allocation between suites of adult phenotypic traits has been well studied under controlled laboratory conditions in several organisms including butterflies (e.g. Nylin 1992; Boggs and Freeman 2005; Hwang et al. 2008; Gibbs et al. 2011). However, to better understand the fitness implications of phenotypes altered by typical anthropogenic environmental conditions, it is essential to study development *in situ* and to do so by looking simultaneously at a set of fitness-related phenotypic traits (Sultan 2007; Kasumovic 2013).

Here, we explored to what extent several fitness-related traits in adult *Aglais urticae* L. (Small Tortoiseshell) butterflies are influenced by anthropogenic environments, by contrasting larval development in forest – their assumed ‘original’ habitat – and in a ‘recent’ agricultural landscape setting. At the larval stage, *A. urticae* is a specialist feeder on *Urtica dioica* L. (stinging nettle). Although current semi-natural woodlands differ in many ways from original, natural woodlands, the overall abiotic conditions (e.g. temperature and humidity) in semi-natural, managed woodlands resemble much more these original conditions compared to open agricultural landscapes. *U. dioica* is associated with nitrogen-rich sunlit places, and as such, it used to be restricted to woodland gaps and river banks on relatively nitrogen-rich soil (Olsen 1921; Strutek 1997). In recent human history, it has benefited from increased soil nitrogen concentrations, both via atmospheric deposition and via direct application and run-off of fertilizers on farmland. Soil eutrophication has allowed *U. dioica* to become quasi-omnipresent in Western Europe, as its main current habitat includes not only broad-leaved woodland, but intensive farmland too (Taylor 2009). The current levels of biologically available nitrogen are well above historic levels and are assumed to have crossed a biophysical threshold with serious consequences for humanity (Rockström et al. 2009). For instance, widespread eutrophication is causing severe biodiversity alterations, including declines for species experiencing raised mortality levels when exposed to elevated nitrogen levels in host plants (Fischer and Fiedler 2000) and for species experiencing microclimatic cooling due to nitrogen-fuelled excessive vegetation growth (Wallis de Vries and van Swaay 2006). In contrast, Betzholtz et al. (2013) have recently shown that this increased availability of nitrogen-rich habitat may be an important driver of range expansions for monophagous Lepidoptera species with a nitrogen-favoured larval diet, such as *A. urticae*.

However, it is not simply the quantitative aspect of the host plant (i.e. abundance and distribution) that matters, but resource quality too. Larval ecological resources include consumables (i.e. leaf quality) and utilities (i.e. microclimate) at the level of the host plant that affect both larval growth and adult trait expression. Local environmental conditions affect host plant quality (e.g. nitrogen content) and thus nutritional value for larvae. But local environmental conditions may also affect larvae independent of host plant quality. For instance, temperature and light intensity during larval development are environmental cues that may trigger phenotypic shifts in both larvae and adults (e.g. Simpson et al. 2011). Solar radiation intensity, ambient temperature and convective cooling are significant microclimatic aspects for the metabolism of ectothermic caterpillars and butterflies (Heinrich 1993; Tattersall et al. 2012). Whilst nettles may typically receive more sunlight and hence reach higher temperatures in field margins than in woodland gaps, woodlands typically provide higher and more buffered levels of soil humidity. However, predicting differences in food plant quality between both environments is not straightforward. Although soil nitrogen levels can be high in field margins, woodland nettles were found to make more growth in terms of dry mass of the shoots than farmland nettles (Taylor 2009). *Urtica dioica* is a shade-tolerant plant, but appears to be very plastic in response to environmental conditions (Pollard and Briggs 1982). All else being equal, warmer and sunnier conditions should result in higher C/N ratios in host plants (Alonso and Herrera 2000), and C/N-ratio shifts may strongly affect plant–herbivore interactions, although species-specific physiological differences in insect metabolic response to foliar C and N exist (Throop et al. 2004). This specificity complicates simple predictions, but there clearly is a need to better explore differences in host plant quality between different habitats in anthropogenic landscapes. At the level of microclimatic differences (independent of nettle leaf quality), we can predict more humid and cooler conditions in woodland compared with field margins (Raich and Tufekcioglu 2000; Merckx et al. 2008).

We used a split-brood design including field margins and woodland gaps so as to study (i) the effect of habitat on phenotypic plasticity by analysing variation in survival, development time, adult body mass and wing morphology and (ii) the heritable basis of the variation in these fitness-related traits. This ecological ‘evo-devo’ approach (Kinnison and Hairston 2007) allows us to obtain insights into how *A. urticae* deals with contemporary anthropogenic environments. Larval environmental differences were validated at the level of both host plant nutritional quality (i.e. C/N ratio) and host plant microclimate (i.e. ambient temperature). Adult body mass is considered to be a proxy of potential reproduction (Karlsson and Van Dyck 2009), whilst wing morphology is indicative of particular aspects of movement ability, with wing loading and aspect ratio particularly closely linked to flight power, capacity and manoeuvrability (Shreeve et al. 2009). Nevertheless, butterfly wings have a range of interacting functions, such as flight, but also thermoregulation, camouflage and intraspecific signalling, which may all be subject to different selection pressures (Shreeve et al. 2009).

Specifically, we predict higher ambient temperatures at field margins to lead to reduced development times. Shorter development may in turn increase survival rates due to shortened exposure to killing agents, such as predators, fungi and parasitoids, which furthermore may be less abundant in these relatively homogeneous biotopes (e.g. Thies et al. 2003). We explore the effects of environmental differences between the two habitat types on adult butterfly trait expression including size, flight-related wing morphology and wing pigmentation. Hence, this allows us to test to what extent the use of the same species of host plant, but under different environmental conditions, may affect larval survival and functional phenotypic design in this common butterfly. For wing pigmentation, we tested the thermal melanism hypothesis (e.g. Clusella Trullas et al. 2007), which predicts higher pigmentation levels under cooler conditions (i.e. under woodland conditions in our case).

## Materials and methods

### Study species

*Aglais urticae* is a Eurasian nymphalid butterfly whose larvae are specialist feeders on nettles, whereas the mobile and colourful adults feed on nectar obtained from a variety of flowers. Adults hibernate, emerging during early spring to mate and breed. The females deposit their eggs in batches on the underside of top leaves of typically young and sunlit nettle shoots, with larvae feeding gregariously under the cover of a jointly spun web till the final larval stage, followed by pupation and eventually eclosion of the next adult generation (Bryant et al. 2000).

### Split-brood experiment

Fifty adult females, wild-caught within one area (Beauraing, Namur, Belgium) during June 2012, were placed for 48 h in individual cages that contained potted nettles for oviposition. Nettle plants were reared by an organic plant nursery and were of similar quality, and each butterfly had access to a water/honey solution. We then collected the complete egg batches of seven females and placed the eggs – separately for each female – upon moist cotton in Petri dishes, under standardized conditions (21°C; 16-h L:8-h D photoperiod) (Bryant et al. 1997, 2000). Just before the eggs hatched, they were transferred to seven larger plastic boxes, with netting on top. In these boxes, the hatched larvae were fed freshly cut nettle leaves, of similar quality, until larvae were 1 cm long.

As a result of this whole procedure, we were able to set up a split-brood experiment with individuals that had so far experienced identical and standardized ‘common garden’ conditions in the laboratory. For each female, 90 larvae were selected, which were split into six groups of 15 larvae each (grand total: 42 groups; 630 larvae). Three of these larval groups were then reared at three different sites within an agricultural setting (i.e. field margins) and the remaining three groups at three different sites within a woodland setting (i.e. woodland gaps). Overall, there were eight agricultural sites and six woodland sites, spread over four town districts within a 10 × 80 km area (Namur and Brabant-Wallon provinces, Belgium). At each site, we erected three cylindrical enclosures of nylon netting (height: 140 cm; diameter: 50 cm) each surrounding six to 10 local nettles. A single group of 15 larvae was placed within each of these 42 enclosures.

Enclosures were visited frequently to collect pupae, which were then kept individually in plastic cups, with netting on top, under the same standardized conditions. These pupae were checked daily. The day after eclosion, adults were sexed and weighed (Mettler Toledo-MT5; accuracy ± 0.1 mg). Then, they were stored at −20°C.

### Development

At each site, ambient temperature was automatically recorded every 15 minutes at the level of the host plant (ca. 130 cm) by a thermoprobe connected to a data logger (HOBO U23-001 Pro v2 Temp/RH, Onset). Upon introducing the larvae into the enclosures, we sampled a nettle leaf from each site for C/N ratio analysis. A green leaf of the apical zone of the plant (second node from the top) was cut, placed in a perforated Eppendorf tube and immediately immersed in liquid nitrogen. Samples were stored at −80°C. Prior to analysis, all samples were dried in an incubator (24 h at 60°C) and then ground using a micro-pestle in liquid nitrogen. C/N ratio analyses were carried out with 6 mg of dried powdered material per leaf using a HCN analyzer (Flash EA 1112 NC Soil Analyzer; Thermo Fisher Scientific Inc., Waltham, MA, USA) measuring the percentage of nitrogen and carbon (Fischer and Fiedler 2000).

Survival was calculated as the percentage of individuals that emerged as adults from the total number of larvae bred in each environmental setting. Development time was measured as the number of days between the hatching of caterpillars and adult emergence.

### Phenotypic traits

Prior to phenotypic measurements, the frozen adults were dried for 24 h to constant body mass in a 60°C incubator. They were then weighed (Mettler Toledo-MT5; accuracy ± 0.001 mg) before the wings were carefully removed from the thorax. Next, scanned images were taken of the dorsal and ventral wing surfaces (Epson Perfection V500 Photo Scanner). Forewing area (cm^2^) and length (cm) were measured on these scans using image analysing software (Image J 1.43u; http://rsb.info.nih.gov/ij/). Wing loading was calculated as body mass/forewing area and aspect ratio as 4 × forewing length^2^/forewing area (Merckx and Van Dyck 2006). Degree of pigmentation was measured as the average grey value (0 = black; 255 = white) of a 0.04-cm^2^ area of each of both dorsal hind wings, with wing scans converted to 8-bit images. Repeated pigmentation measurements revealed a high level of repeatability (i.e. 98.5 ± 0.5%; *N* = 20). We consider the pigmentation of the selected area to be representative of the whole wing's degree of pigmentation.

### Statistical analyses

Differences between the enclosures at agricultural and woodland habitats in host plant nutrient contents and ambient temperatures were tested with generalized linear models. Development time, body mass and wing traits were tested using generalized linear mixed models, in which we tested each of the dependent variables relative to environmental setting (i.e. agricultural versus woodland), sex, and their interaction, with ‘family ID’ as a covariate. These tests were likelihood ratio tests (LRT), which follow the chi-square distribution with one degree of freedom. In addition, development time was added as a covariate to the body mass and wing traits models. ‘Enclosure ID’ nested within ‘site ID’ and ‘site ID’ nested within ‘town ID’ were added as random factors. As agricultural and woodland settings differed significantly in host plant quality (i.e. C/N ratio) and host plant microclimate (i.e. ambient temperature near host plants) (see Results), we added another set of analyses in which we replaced ‘environmental setting’ with the factors ‘host plant quality’ and ‘temperature’, but now with the analyses separated by environmental setting. All analyses were performed in R (R2.14.1; package lme4; http://lib.stat.cmu.edu/R/CRAN).

## Results

### Development

#### Host plant quality and temperature

Mean ambient temperatures of field margin nettles were on average 1.4°C higher (LRT *χ*²_1_ = 8.57, *P *=* *0.0034), and more variable, than woodland gap nettles (Table1). This elevated variance in mean temperatures at field margins is reflected in the on average 1.2°C lower minimum temperatures (LRT *χ*²_1_ = 4.57, *P *=* *0.033), and especially in the on average 8.1°C higher maximum temperatures (LRT *χ*²_1_ = 15.19, *P *<* *0.0001) at field margins than at woodland gaps (Table1).

**Table 1:** Summary statistics of host plant minimum, average and maximum temperature for field margins (F; *N* = 8) versus woodland gaps (W; *N* = 6)

	Minimum temp. (°C)		Average temp. (°C)		Maximum temp. (°C)	
	F	W	F	W	F	W
Mean ± SE	5.6 ± 0.4	6.8 ± 0.5	17.1 ± 0.5	15.7 ± 0.1	36.9 ± 1.8	28.8 ± 1.0
Min.	3.9	5.1	15.2	15.4	31.6	26.7
Max.	7.3	8.4	19.0	16.1	44.3	33.5

Although the nitrogen content of nettles did not differ significantly between field margins and woodland gaps (LRT *χ*²_1_ = 0.89, *P *=* *0.34), field margin nettles had on average a higher carbon content than nettles of woodland gaps (LRT *χ*²_1_ = 5.45, *P *=* *0.020), resulting in a 15% higher C/N ratio (LRT *χ*²_1_ = 4.35, *P *=* *0.037), whilst the range between the maximum and minimum C/N ratio was 55% larger at field margins than at woodland gaps (Table2).

**Table 2:** Summary statistics of host plant nitrogen percentage, carbon percentage and C/N ratio for field margins (F; *N* = 8) versus woodland gaps (W; *N* = 6)

	Nitrogen (%)		Carbon (%)		C/N	
	F	W	F	W	F	W
Mean ± SE	4.1 ± 0.3	4.5 ± 0.3	39.7 ± 0.6	37.7 ± 0.7	9.9 ± 0.5	8.6 ± 0.4
Min.	3.2	3.5	37.8	35.4	7.2	7.2
Max.	5.9	5.5	42.3	39.5	11.8	10.1

#### Survival and development time

**Figure 1:**
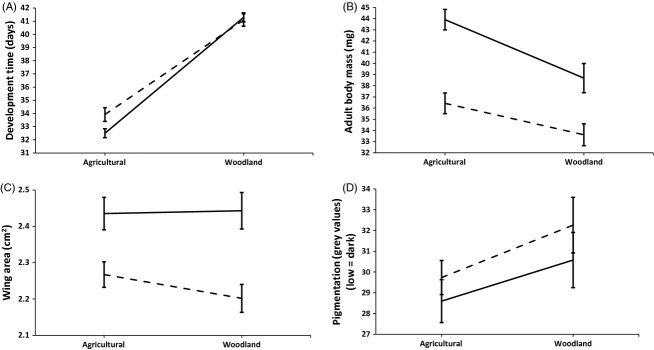
Mean values (±SE) of (A) juvenile development time (i.e. larval + pupal development), (B) dry body mass, (C) forewing area and (D) wing pigmentation for both female (solid line) and male (dashed line) *Aglais urticae* individuals that developed in agricultural versus woodland settings.

Overall survival was 15.2%, with survival rates tending to be higher in agricultural than in woodland settings [18.1% (57/315) vs 12.4% (39/315), respectively; Fisher's exact test: *P *=* *0.059]. Larvae developed ca. 20% faster in field margins than in woodland gaps (LRT *χ*²_1_ = 16.7, *P *<* *0.001) (Fig.1A), with no differences in overall development time between males and females (LRT *χ*²_1_ = 0.11, *P *=* *0.73). Development times differed significantly among families (LRT *χ*²_6_ = 13.52, *P *=* *0.03). Within the temperature range recorded in field margins, only females reduced development time with increasing temperature (i.e. by ca. two days over a 15–20°C range; Temperature x Sex: LRT *χ*²_1_ = 5.92, *P *=* *0.01).

### Phenotypic variation

#### Body mass and wing area

Butterflies that developed in field margins were ca. 13% heavier than those that developed in woodland gaps (LRT *χ*²_1_ = 8.10, *P *=* *0.004), with females heavier than males (LRT *χ*²_1_ = 18.85, *P *<* *0.001) (Fig.1B). Temperature and host plant quality did not affect body mass within both developmental settings (*P* > 0.56).

**Figure 2:**
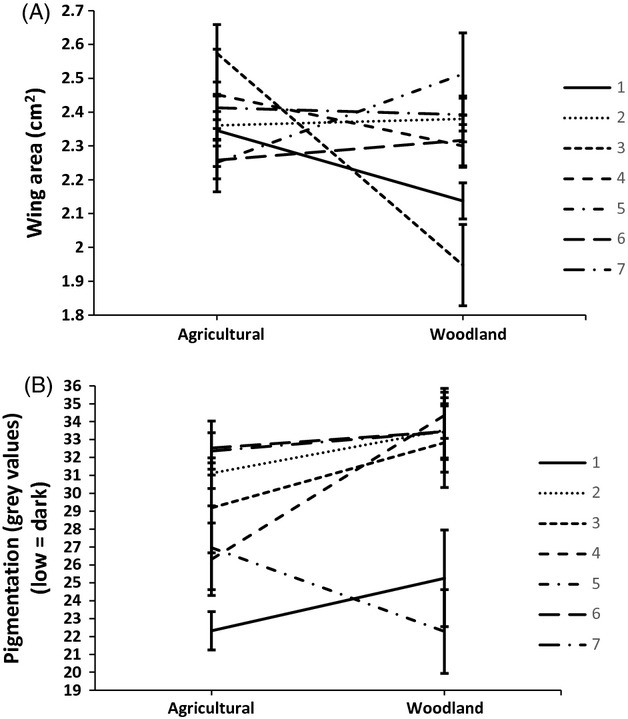
Mean values (±SE) of (A) forewing area and (B) wing pigmentation for the adult offspring of the seven families in both agricultural and woodland developmental habitats.

Butterflies that developed in field margins had significantly larger wings than those that in woodland gaps (LRT *χ*²_1_ = 16.68, *P *<* *0.001), with females having larger wings than males (LRT *χ*²_1_ = 40.47, *P *<* *0.001) (Fig.1C). Although individuals that developed in field margins were on average larger-winged, this was not the case for every family; whilst we observed significant variation in wing length among families overall (LRT *χ*²_6_ = 13.53, *P *=* *0.03), some families produced offspring of similar wing length in both habitats, and one family completely bucked the trend, producing larger-winged individuals in woodland gaps than in field margins (Family x Habitat: LRT *χ*²_6_ = 23.82, *P *<* *0.001) (Fig.2A). Within both developmental settings, temperature did not affect wing area (*P* > 0.95). Host plant quality did affect wing area, but only so in the agricultural setting and only for females (C/N × Sex: LRT *χ*²_1_ = 5.11, *P *=* *0.024), which grew smaller wings with poorer plant conditions (i.e. higher C/N).

#### Wing loading and aspect ratio

Neither wing loading nor aspect ratio did differ between developmental settings (*P* > 0.52).

#### Wing pigmentation

Butterflies bred in field margins had significantly darker wings than those bred in woodland gaps (LRT *χ*²_1_ = 7.6, *P *=* *0.006) (Fig.1D). Significant variation among families was present (LRT *χ*²_6_ = 35.9, *P *<* *0.001) and the same family that bucked the overall trend regarding wing area did so regarding wing pigmentation too, producing darker-winged individuals in woodland gaps than in field margins (Fig.2B). Neither temperature, nor host plant quality, nor sex did affect wing pigmentation within the agricultural setting (*P* > 0.39). However, within the woodland setting, males, but not females, developed darker wings under both warmer and lower host plant quality conditions, with males being paler than females at relatively low developmental temperatures and at relatively high host plant quality (i.e. low C/N), but being darker than females at relatively high temperatures and at relatively low host plant quality (i.e. high C/N) (Sex: LRT *χ*²_1_ = 7.3, *P *=* *0.007; Temperature x Sex: LRT *χ*²_1_ = 12.9, *P *<* *0.001; C/N × Sex: LRT *χ*²_1_ = 10.28, *P *=* *0.001).

## Discussion

Nettle-feeding insects such as *A. urticae* occur in several environments including woodland and agricultural landscapes, but depending on the landscape context, host plants and local microclimates have different consequences for larval survival and adult phenotypic expression. Our split-brood approach indicated plastic responses within families, but also family effects in the phenotypic responses. *A. urticae* developed 20% faster at field margins compared with woodland gaps. This observation is best explained by the higher ambient temperatures at field margins than at woodland gaps. Ambient temperature being a key factor for development rates, we showed that females developing in the warmest field margins did so two days faster than females in the coolest field margins. We predicted that the shorter development time in field margins would increase survival rates due to shortened exposure to killing agents; survival rates did increase, from 12% to 18%, though only near significantly so.

These increased development rates, and associated near-significant improvements of survival rates, occurred despite the on average 15% higher C/N ratios (i.e. presumed lower nutritional quality) of field margin nettles. Nevertheless, leaf nitrogen percentages *per se* did not differ statistically between field margins and woodland gaps, and it is known that larvae are able to compensate N accumulation rates, within limits, for reduced nitrogen content by eating more food, and doing so faster, and/or by selecting the most nitrogen-rich plant parts (Slansky and Feeny 1977; Obermaier and Zwölfer 1999). As such, to tease apart the presumed nutritional quality and microclimate effects, we call for a laboratory experiment in which *A. urticae* development rates are scored for several C/N ratio regimes, whilst keeping leaf nitrogen percentages constant, and this under a few temperature regimes. Also, apart from C and N, several other components of host plant quality, such as trace elements and defensive compounds, are known to potentially impact larval development in herbivorous insects (Awmack and Leather 2002), and these may have differed between both habitat types.

Although we observed differences in the average environmental conditions under which the caterpillars developed in both habitats, we also noticed important differences in the variance of those conditions. Our key observation is that the anthropogenic field margin environment creates a broader phenotypic space (e.g. Pigliucci 2007) for *A. urticae*; whereas its host plant used to be restricted to the relatively buffered woodland environment, anthropogenic eutrophication has meant that the host plant now occurs in more exposed and hence microclimatically more variable settings outside woodlands too. For example, the standard error around the mean temperature was almost five times larger at field margins than at woodland gaps. In turn, this may also affect variation in soil moisture, a variable related to the uptake of available soil nitrogen by plants and storage into their leaves (Sprent 1976). More variable soil moisture levels at field margins (Raich and Tufekcioglu 2000) may explain the larger variability in nettle leaf C/N ratios observed in field margin nettles. Our experiment suggests that this variability in resource quality – of leaf nutritional value and microclimate – both within field margins and between woodlands and field margins has led *A. urticae* to occupy a larger phenotypic parameter space overall than the phenotypic variability realized originally within woodlands alone. Indeed, as butterflies that developed in field margins were on average heavier and had larger and darker wings than those that developed in woodland gaps, the total phenotypic space currently occupied by the species is likely to be larger than its ‘original’ phenotypic space realized under woodland conditions only. An assessment of the precise dimensions of this added phenotypic space under natural conditions is now warranted. Whilst we forced larvae to develop on specific nettle plants – although *in situ* – these plants may or may not have been selected by ‘choosy’ ovipositing females who may well adapt host plant choice in line with (anticipated) host plant quality and (anticipated) host plant environment (Thompson and Pellmyr 1991; Awmack and Leather 2002). It would thus be interesting (i) to compare the quality of plants chosen for oviposition by female *A. urticae* versus host plants without eggs and (ii) to assess the phenotypic dimensions of adult offspring bred on host plants naturally selected by their mothers within field margin and woodland gap environments.

Our results on wing pigmentation showed significant habitat-specific differences in both males and females. Based on work on *Drosophila*, phenotypic plasticity of pigmentation has recently been proposed to be a side effect reflecting the impact of temperature on epigenetic mechanisms (Gilbert et al. 2007). Darker pigmentation observed at low developmental temperatures (e.g. in *Drosophila*: Gilbert et al. 2000) has traditionally been assumed to be an adaptive plasticity (i.e. thermal melanism hypothesis, which sees the production of higher levels of dark pigments during development as an adaptive response to colder conditions by increasing thermal energy absorption as larvae or adults: Davis et al. 2005; Clusella Trullas et al. 2007). However, we found the opposite pattern as darker pigmentation was observed in butterflies that developed in warmer field margins compared to cooler woodland. But besides the thermal melanism hypothesis, there is also a less frequently explored hypothesis on the positive relationship between desiccation resistance and pigmentation (e.g. Parkash et al. 2008). Higher levels of pigmentation under higher desiccation risks are in line with the observed pattern in our data. This warrants further mechanistic research. Lower nitrogen levels in drought-stressed host plants were associated with lower pigmentation in the butterfly *Pararge aegeria* (Talloen et al. 2004), but there were no significant differences in nitrogen between the habitats in our study. Besides direct microclimatic conditions or host plant quality effects, changes in pigmentation may also result from correlated changes in other life-history traits (Roff and Fairbairn 2013). Wing pigmentation is a complex phenomenon at both the proximate and ultimate level (True 2003), but our results show scope for interesting work in the context of anthropogenic landscapes.

The increased development rate, increased tendency for survival and larger phenotypic space of *A. urticae* bred in field margins may help this nettle-feeding butterfly species to deal successfully with human-induced rapid environmental changes. Although only a detailed study into fitness consequences could clarify whether or not these observed patterns are evolutionary adaptive, our findings contrast with parallel work on *A. io* L. (Peacock Butterfly). For this phylogenetically and ecologically closely related nettle-feeding species, these anthropogenic biotopes may pose a conflict for choosing what is ultimately the best breeding habitat, as more, but smaller offspring is produced in woodland gaps, whereas less offspring, but of better quality, is produced in field margins (Serruys and Van Dyck 2014). Our results for *A. urticae* did not show such a trade-off, as both more and larger offspring were produced in field margins. In contrast to the univoltine *A. io*, *A. urticae* has several generations each year, and such evolved and elevated temporal plasticity in development and design not only allows this species to respond to seasonally changing conditions, but may also have facilitated adaptive phenotypic plasticity in response to anthropogenic environmental change (Ishihara 1999; Merckx and Van Dyck 2006; Van Dyck et al. 2009).

*Aglais urticae*'s successful exploitation of nettles outside the woodland environment may be more common given the range expansions in recent decades for monophagous moth and butterfly species with a nitrogen-favoured larval diet (Betzholtz et al. 2013). Nevertheless, although nettle-specialists, with mobile and fast-developing phenotypes selected in response to the historical patchy and ephemeral occurrence of their host plant (Wallis de Vries 2014), may nowadays find host plants much more frequently in the eutrophic landscape matrix between woodland fragments, several biotic (e.g. parasitoid impact) and abiotic conditions are likely to be more variable. For instance, droughts and predicted climatic change may reduce host plant quality and hence breeding success of *A. urticae* more strongly in exposed fields than in buffered woodlands (Pollard and Greatorex-Davies 1997; Settele et al. 2008). The elevated variability of this evolutionary novel environment may hence explain the strong fluctuations in abundance from year to year for this species (e.g. Van Dyck et al. 2009); whilst the larger size of individuals bred in field margins may positively influence potential fecundity (Karlsson and Van Dyck 2009), their realized fecundity and fitness are probably set to differ largely among years. Further work comparing fitness-related parameters in both biotopes among years should ideally also include immune-related parameters, as increased ambient temperatures may decrease immune function, although such an effect is likely to be further modulated by food stress conditions too (Karl et al. 2011). Although we focused on the effects of host plants in different habitats, other resources may also affect these butterflies in changing human-dominated landscapes. The general declines in abundance and distribution of wild flowers, and hence nectar supply, across homogenized landscapes (Tscharntke et al. 2005) may lower fecundity (O'Brien et al. 2004). Several studies indicated recent population declines in several common butterflies, including *A. urticae* (Van Dyck et al. 2009; Wallis de Vries et al. 2012; Botham et al. 2013), despite some weather-related annual increases in abundance, and despite the abundance of their host plant resource.

Given projected land-use and climatic change, it is important to understand how rapid human-induced environmental change affects development, trait expression and evolution of species (Sih et al. 2011; Kasumovic 2013) and to understand which characteristics predispose species to become either ‘winners’ or ‘losers’ under anthropogenic change.

## Data archiving statement

Data for this study are available at the Dryad Digital Repository: http://dx.doi.org/10.5061/dryad.2c972.
